# Establishing a health information workforce: innovation for low- and middle-income countries

**DOI:** 10.1186/1478-4491-11-35

**Published:** 2013-07-18

**Authors:** Jenny H Ledikwe, Letitia L Reason, Sarah M Burnett, Lesego Busang, Stephane Bodika, Refeletswe Lebelonyane, Steven Ludick, Ellah Matshediso, Shreshth Mawandia, Mpho Mmelesi, Baraedi Sento, Bazghina-werq Semo

**Affiliations:** 1 Department of Global Health, University of Washington, Seattle, Washington, USA; 2 Botswana International Training and Education Center for Health (I-TECH), Gaborone, Botswana; 3 Accordia Global Health Foundation, Washington, DC, USA; 4 African Comprehensive HIV/AIDS Program, Gaborone, Botswana; 5 United States Centers for Disease Control and Prevention, Gaborone, Botswana; 6 Botswana Ministry of Health, Gaborone, Botswana; 7 Botswana Ministry of Local Government, Gaborone, Botswana; 8 University of Botswana, Gaborone, Botswana; 9 Tanzania International Training and Education Center for Health (I-TECH), Dar es Salaam, Tanzania; 10 Botswana National AIDS Coordinating Agency, Gaborone, Botswana

**Keywords:** Health information, Monitoring and evaluation (M&E), Data quality, Task shifting, Health workforce

## Abstract

**Background:**

To address the shortage of health information personnel within Botswana, an innovative human resources approach was taken. University graduates without training or experience in health information or health sciences were hired and provided with on-the-job training and mentoring to create a new cadre of health worker: the district Monitoring and Evaluation (M&E) Officer. This article describes the early outcomes, achievements, and challenges from this initiative.

**Methods:**

Data were collected from the district M&E Officers over a 2-year period and included a skills assessment at baseline and 12 months, pre- and post-training tests, interviews during stakeholder site visits, a survey of achievements, focus group discussions, and an attrition assessment.

**Results:**

An average of 2.7 mentoring visits were conducted for M&E Officers in each district. There were five training sessions over 18 months. Knowledge scores significantly increased (*p* < 0.05) during the three trainings in which pre/post tests were administered. Over 1 year, there were significant improvements (*p* < 0.05) in self-rated skills related to computer literacy, checking data validity, implementing data quality procedures, using data to support program planning, proposing indicators, and writing M&E reports. Out of the 34 district M&E Officers interviewed during site visits, most were conducting facility visits to review data (27/34; 79%), comparing data sets over time (31/34; 91%), backing up data (32/34; 94%), and analyzing data (32/34; 94%). Common challenges included late facility reports (28/34; 82%), lack of transportation (22/34; 65%), inaccurate facility reports (10/34; 29%), and colleagues’ misunderstanding of M&E (10/34; 29%). Six posts were vacated in the first year (6/51; 12%). A total of 49 Officers completed the achievements survey; of these, common accomplishments related to improvements in data management (35/49; 71%), data quality (31/49; 63%), data use (29/49; 59%), and capacity development (26/49; 53%).

**Conclusions:**

The development of a cadre of district M&E Officers has contributed positively to the health information system in Botswana. In the absence of tertiary training related to health information, on-the-job training and mentoring of university graduates can be an effective approach for developing a new professional cadre of M&E expertise and for strengthening capacity within a national health system.

## Background

Many low- and middle-income countries experience critical shortages of health workers and imbalances in the mix of skills within the health workforce [1,2], a challenge exacerbated by the HIV/AIDS epidemic [3]. This widely recognized global health problem impedes efforts to reach overall health goals [4,5]. Discussions and interventions on health worker shortages often focus on the need for physicians and other clinical staff who deliver prevention, care, and treatment services [5-7]. However, to maintain a well-functioning health system, health care providers need to be complemented by nonclinical personnel with additional relevant skills [5]. This includes health workers who monitor and evaluate health program performance to ensure effective and efficient implementation. The World Health Organization has identified health information systems as one of the six core components or “building blocks” of a health system [8].

Recognition of the importance of health information systems is growing, partially due to unprecedented donor assistance and global health initiatives such as the United States President's Emergency Plan for AIDS Relief (PEPFAR), the Bill and Melinda Gates Foundation, and the Global Fund to Fight AIDS, Tuberculosis, and Malaria [8]. As health programs expand to address HIV/AIDS and related morbidities, there have been concomitant increases in the amount of data generated due to the rising numbers of health programs, service delivery sites, and patients [9]. Similarly, the number of indicators being monitored within health information systems has increased, and there is greater emphasis on accountability and transparency in program budget execution. Even though the importance of data is being increasingly recognized, poor data quality has been noted in a number of low- and middle-income countries [10-18], with human resource challenges being a contributing factor [9,10,12,15,16,19,20].

In Botswana, human resource constraints were identified as one factor adversely affecting the health information systems as part of the *2010 Botswana Country Report on Progress Toward the 2001 Declaration of Commitment on HIV and AIDS*[19]. The lack of health informatics as well as monitoring and evaluation (M&E) training programs within tertiary institutions in Botswana has contributed to this situation. Additionally, health-care workers who traditionally assumed data-related responsibilities have been challenged to fulfill these duties because of the increasing workload associated with health program expansion. To rapidly address the need for health information personnel, a task-shifting approach was implemented in which a new cadre – district M&E Officers – was created. Task shifting is a strategy to help alleviate health workforce shortages by delegating tasks to new or existing cadres [1]. Since individuals trained in health informatics or M&E were not available for recruitment, an innovative approach was undertaken to build this new cadre: university graduates in the field of social sciences were recruited and provided with on-the-job training and mentoring in order to provide them with the knowledge and skills necessary to carry out M&E responsibilities. A total of 51 district M&E Officers were initially employed, with most of the 27 districts in the country receiving two officers. They were placed within the District Health Management Teams and the Offices of the District AIDS Coordinator. While supervision differed by district, the members of the new cadre were generally supervised by a Public Health Specialist, a Community Health Nurse, or the District AIDS Coordinator. All officers were from Botswana and were initially hired on a fixed-term (1–3 year) contract. The role of the new cadre was multifaceted, with an expectation that they would assume most data-related activities previously conducted by a range of health workers. The scope of work for the new cadre focused on supporting health facilities, nongovernmental organizations, and district-level entities in strengthening health data collection, ensuring regular and timely reporting and feedback, promoting a culture of data utilization and evidence-based planning, and building capacity within the health system. Additionally, the district M&E Officers would be responsible for compiling district-wide health data and reporting to the national programs. The reports generated by the M&E Officers were to be used to strengthen and inform programmatic planning at facility, district and national level.

Multiple stakeholders collaborated on this initiative. The Ministry of Local Government (MLG) and Ministry of Health (MOH) were responsible for administrative and technical oversight of the district M&E Officers. The US Centers for Disease Control and Prevention (CDC) and the African Comprehensive HIV/AIDS Partnership (ACHAP) funded salaries of the new cadre and provided technical guidance in conjunction with UNAIDS and the National AIDS Coordinating Agency. The International Training and Education Center for Health (I-TECH) in Botswana provided training and mentoring support.

Trainings were conducted two to three times a year and included skill-building workshops and didactic sessions. On-site mentoring visits lasted 1 to 2 days with the purpose of reinforcing knowledge and skills gained during trainings as well as troubleshooting other work-related challenges. Mentoring was tailored to the individual needs of the District M&E Officers and was conducted by M&E Officers from I-TECH in conjunction with M&E staff from the Ministry of Local Government. At the beginning of the project, an exit strategy was developed to ensure sustainability beyond donor support; this included a focus on ensuring that all activities related to the cadre were overseen by staff within the Government of Botswana and ensuring that discussions related to the absorption of the cadre in the Government of Botswana public health system were held frequently throughout development an initiation of the cadre. The objective of this article is to describe the early outcomes, achievements, and challenges of the new cadre.

## Methods

A mixed methods approach with qualitative and quantitative data was used. The data were collected from the district M&E Officers over a 2-year period (Table 1) and included a skills assessment at the beginning of the cadre’s tenure and after 12 months, pre- and post-training tests, interviews during stakeholder site visits, a survey focusing on achievements, focus group discussions, and an attrition assessment. These data were collected for monitoring program implementation. Use of the data for this publication was approved by the Botswana Health Research and Documentation Committee, the ethical review board overseeing human health research at the Ministry of Health. While there were 51 district M&E Officers hired at the outset, the number of occupied positions varied over the course of the project as some Officers left during the course of implementation while others were also recruited into the cadre.

**Table 1:** Timeline for data collection activities

**Methodology**	**Data collection timing**				
	**Baseline**	**6 months**	**12 months**	**18 months**	**24 months**
Skills assessments	X		X		
Pre- and post-training tests		X	X	X	
Interviews during stakeholder site visits				X	
Survey of achievements				X	
Focus group discussions					X
Attrition assessment			X		

To create an initial profile of capabilities and to ascertain training needs, baseline self-assessments were completed by 40 district M&E Officers, representing 78% (40/51) of the posts. While focused on M&E skills, the assessment also included items about educational background, with a 5-point Likert scale used to self-report skill levels. The same assessment tool was re-administered after 12 months with 31 of the original 40 respondents completing the tool (78%).

Pre- and post-training tests were administered at three of the five training sessions for the district M&E Officers, which were conducted at approximately 6, 12, and 18 months after the initiation of the cadre. This included trainings on the national HIV/AIDS programs, data management, and data analysis, with 30, 31, and 43 M&E Officers completing pre-post training tests at each of these trainings, respectively. Changes in knowledge were assessed using multiple choice and true/false questions.

Interviews were conducted during site visits by stakeholders at 18 months to collect information on the activities being conducted by the district M&E Officers. Stakeholders included representation from MLG, MOH, UNAIDS, NACA, CDC, ACHAP, and I-TECH. Thirty-four of the district M&E Officers, which represents 67% (34/51) of the posts, were interviewed during these visits using a standardized questionnaire with open-ended questions.

After 18 months, 49 district M&E Officers, representing 96% of the posts (49/51), completed a brief, written survey to provide information on their experiences as district M&E Officers. The survey included open-ended questions that focused on achievements. The survey was administered in-person to the participants while they were attending a workshop. The instructions were read to participants as a group, and they were provided with an opportunity to ask questions or seek clarity if they felt they needed assistance.

Two focus group discussions were conducted with the district M&E Officers after 24 months to learn more about the capacity building efforts undertaken to develop the cadre. Officers within approximately 40 km of the two main city centers were invited to participate in the focus groups. A total of 23 district M&E Officers participated, representing 45% of the posts within the cadre (23/51). Focus group discussions were recorded, transcribed, and translated (where necessary) before analysis.

Approximately 1 year after the initiation of the project, telephone interviews were conducted with all six district M&E Officers who had left their positions during that year. This attrition assessment included open-ended questions focusing on their reasons for resignation.

### Data analysis

Quantitative data were analyzed using SPSS (PASW Statistics for Windows, Version 18.0.0, SPSS, Inc.). This included data from the knowledge and skills assessment administered at baseline and 12 months as well as pre- and post-training test scores. Data were summarized using descriptive statistics, with paired t-tests performed to assess changes in knowledge and self-rated skill. A general inductive approach was taken for analyzing the qualitative evaluation data from the survey, focus groups, and stakeholder site visit reports [21]. This involved the manual coding of textual data and identification of common themes in order to condense the data into a summary format and establish links with the evaluation objectives.

## Results

### Baseline skills assessment

Data from the baseline skills assessment (*n* = 40) indicate that 55% (22/40) of the district M&E Officers were female. All respondents held a Bachelors’ degree, with the most common fields of study being economics, statistics, and demography. When asked to list health or statistics courses they had taken, most (30/40; 75%) reported they had taken at least one university-level course in statistics; however, few (6/40; 15%) reported taking a health course.

Respondents rated their computer skills and various professional skills. While most (29/40; 73%) indicated they had experience with Microsoft Word, less than half of the Officers indicated they were experienced with Microsoft Excel (17/40; 43%), PowerPoint (8/40; 20%), or Access (5/40; 13%). The baseline assessment also included five basic multiple-choice questions related to HIV knowledge. All respondents correctly indicated that PMTCT was an acronym for prevention of mother-to-child-transmission of HIV. Most identified that CD4 counts were used to assess antiretroviral (ARV) therapy eligibility among HIV patients (38/40; 95%) and knew that isoniazid preventive therapy was used to prevent tuberculosis (36/40; 90%). Approximately 80% were able to identify the purpose of the national ARV program (33/40; 83%). A quarter of the Officers (10/40; 25%) were able to correctly identify that nevirapine is commonly used for prevention of mother-to-child transmission of HIV. When presented with simple data sets, the majority was able to correctly calculate a median (38/40; 95%), a mean (34/40; 85%), and a percent (34/40; 85%); draw a graph (32/40; 80%); and complete a simple data aggregation exercise (37/40; 93%).

### Training and mentoring

Data from the baseline skills assessment were used to develop a training and mentoring plan for the district M&E Officers. Over the 2-year period following the initiation of the cadre, seven trainings were conducted specifically for the district M&E Officers (Table 2). Pre- and post training tests were administered during three of these trainings. There were significant increases (*p* < 0.05) in average knowledge scores for each of the three trainings. Mentoring visits were conducted at the M&E Officers workplace to address day-to-day technical issues and reinforce concepts presented at the trainings. On average, 2.7 visits were made to each of the 27 districts (range = 1–5) over the 2 years.

**Table 2:** Trainings held for M&E Officers over 18 months with pre-test and post-test score, when collected

	**Training topic**	**Date**	**Description**	**Average pre-test score**	**Average post-test score**
1	Health information management systems (HIMS)	Oct 2007	5-day training to improve awareness, understanding, and technical skills of the M&E Officers in relation to the creation, acquisition, processing, dissemination, and use of quality health data	N/A	N/A
2	M&E workshop	Nov 2007	5-day training to develop and strengthen the capacity of the M&E Officers to monitor and evaluate HIV/AIDS programs. The workshop covered five main areas: general M&E information, M&E frameworks, indicators and data sources, evaluation, and utilization of M&E information	N/A	N/A
3	National HIV/AIDS programs	Apr 2008	6-day training providing an overview of national health programs administered throughout the country (e.g., HIV treatment, sexually transmitted infections, TB, HIV testing and counseling, etc.).	62.5	74.4^**^
4	Data management and utilization	Sep-Oct 2008	5-day training on collecting, managing, analyzing, and interpreting health-care data using DHIS and e-BHRIMS	46.8	70.0^**^
5	Data analysis, report writing, and dissemination	Mar 2009	5-day training on analyzing and disseminating M&E data, enhancing data quality, program planning, and program implementation at the district-level	64.0	78.0^*^

^*^p < 0.05 based on a paired t-test.

^**^p < 0.001 based on a paired t-test.

### Twelve-month skills assessment

Items from the skills assessment administered at baseline were re-administered to the Officers at 12 months (Table 3). There were significant improvements (*p* < 0.05) in self-rated computer literacy during the first year related to use of Microsoft Excel to create tables, to create graphs, and to do calculations; use of a word-processing program to write letters and memos, to create tables, and to insert graphics; and use of Microsoft PowerPoint to create presentations. Skill related to Microsoft Access remained low at 12 months. There were also improvements in self-rated ability to check data validity and implement data quality procedures; use data to support program planning; propose indicators for program monitoring; and write reports to disseminate M&E information. There were no significant changes related to data analysis, knowledge of basic epidemiology, program evaluation, or self-assessment ability.

**Table 3:** Baseline and 12-month skills assessment results (based on a 5-point scale; 1 = no ability, 5 = high ability)

**Topic area**	**Mean baseline ± SD**	**Mean 1 year ± SD**	***n***
Excel:			
Create tables	3.39 ± 0.99	3.97 ± 1.05*	31
Perform calculations	3.27 ± 1.29	4.10 ± 0.89**	30
Create graphs	3.26 ± 1.13	4.13 ± 0.92**	31
Word:			
Write letters/memos	4.10 ± 0.98	4.71 ± 0.59**	31
Format text	4.10 ± 1.01	4.42 ± 0.92	31
Create tables	3.70 ± 1.02	4.67 ± 0.66***	30
Insert graphics	3.15 ± 1.20	4.00 ± 1.33**	27
PowerPoint:			
Create presentations	1.94 ± 1.34	3.97 ± 1.05***	31
Access:			
Input data	1.86 ± 1.13	1.90 ± 1.18	29
Generate reports	1.62 ± 1.02	1.86 ± 1.19	29
E-mail:			
Send e-mails	4.23 ± 1.15	4.45 ± 1.06	31
Team work:			
Effectively communicate	3.61 ± 0.92	3.35 ± 1.02	31
Design projects/programs	3.87 ± 1.04	3.50 ± 1.28	30
Data quality:			
Check validity	3.35 ± 1.02	4.00 ± 1.07*	31
Implement procedures to improve	3.19 ± 1.20	3.90 ± 0.98*	31
Data analysis:			
Interpret data	3.55 ± 0.96	3.87 ± 0.85	31
Aggregate data	3.81 ± 1.11	3.48 ± 1.03	31
Assess data consistency	2.81 ± 1.22	3.10 ± 1.11	31
Use data from more than one source	2.61 ± 1.46	2.81 ± 0.98	31
Epidemiology:			
Knowledge of basics	1.97 ± 1.27	1.97 ± 1.02	29
Assessment:			
Develop relevant questions	2.38 ± 1.24	2.48 ± 0.99	29
Understand different questions and designs get different results	2.27 ± 1.02	2.30 ± 1.02	30
Design strategies & tools	2.34 ± 1.26	2.28 ± 0.88	29
Program planning:			
Support programs to use data	1.73 ± 0.91	2.47 ± 0.97**	30
Monitoring:			
Propose indicators	2.10 ± 1.08	3.00 ± 0.97***	31
Work with program to identify inputs, outputs, and impacts	2.52 ± 1.26	2.90 ± 1.08	31
Evaluation:			
Recommend evaluation questions	2.52 ± 0.96	2.94 ± 1.12	31
Use qualitative and quantitative data	2.61 ± 1.17	2.55 ± 1.00	31
Triangulate data	1.65 ± 0.84	1.71 ± 1.04	31
Support program managers in conducting evaluations	2.16 ± 1.16	2.45 ± 1.03	31
Dissemination:			
Public speaking	2.93 ± 1.11	2.97 ± 1.19	30
Write evaluation report	2.43 ± 1.14	2.87 ± 1.20*	30
Effectively communicate results	2.83 ± 1.20	3.17 ± 1.10	29
Use data for programmatic decision-making	3.14 ± 1.43	3.52 ± 1.18	29
Self-assessment:			
Self-assessmentIdentify personal strengths and weaknesses	3.63 ± 1.16	3.73 ± 1.08	30
Self-assessmentIdentify skills areas needing improvement	3.80 ± 1.13	3.83 ± 1.18	30

*p < 0.05 based on a paired t-test.

**p < 0.05 based on a paired t-test.

***p < 0.001 based on a paired t-test.

### Interviews during site visits

Information related to the duties undertaken by the district M&E Officers was gathered during site visits conducted by stakeholders at 18 months. It was found that all 34 of the district M&E Officers who participated in the site visits were engaged in M&E-related activities; however, almost one quarter of the Officers (8/34; 24%) were also engaged in non-M&E activities such as community mobilization and implementation of HIV programmatic initiatives. Essentially all of the Officers (32/34; 94%) reported being engaged in activities to improve data quality and security. This included conducting facility visits to review data (79%) as well as comparing new data with values previously received (31/34; 91%) and backing up data at regular intervals (32/34; 94%). Most Officers (32/34; 94%) also indicated they were involved in data analysis, including generating routine program reports, compiling data for evidence-based planning activities, and investigating specific health issues such as increases in diarrhea cases or teenage pregnancies.

Challenges identified during the stakeholder visits included late and inaccurate reports from health facilities, which were reported by 82% (28/34) and 29% (10/34) of the district M&E Officers, respectively. Lack of transportation was reported by 65% (22/34) of the Officers as a challenge for data verification and capacity development at facilities. Additionally, 18% (6/34) reported that the electronic data systems were not functioning properly. Furthermore, 29% of those visited (10/34) reported that colleagues and district management were not sensitized to M&E and did not understand its importance, with reports of Program Officers being uncooperative and difficult to work with by 15% of the district M&E Officers (*n* = 5/34).

### Survey of achievements

Further information about the accomplishments of the district M&E Officers was obtained from the survey on achievements, which was completed by 49 Officers. As depicted in Table 4, responses encompassed seven general themes. The most common accomplishments were related to improvements in data management (35/49; 71%), data quality (31/49; 63%), and data use (29/49; 59%). Capacity development activities were listed by a little over half of the Officers (26/49; 53%); many listed accomplishments that fell outside of the expected job duties for the district M&E Officers (21/49; 43%). Reporting duties were seen as accomplishments by ten Officers (10/49; 20%), and five (5/49; 10%) indicated they had been involved in operations research activities.

**Table 4:** **Responses from survey asking District M&E Officers to list the their achievements (*n *= 49)**

**General theme**	**Topic area included**	***Number of survey respondents***	***Selected quotations from respondents***
Data management	Modifying or developing reporting tools, records, and file management at facilities or districts, electronic data storage and back-up, streamlining and coordinating data flow	35	• *“[I] also came up with a reporting format for CBOs [community-based organizations], something which was not there before my arrival”*
			• *“When I arrived in the district there were no data for the past 4 years. I was forced to go to MOH and ask for the data for the previous years”*
			• *“I managed to improve the problem of stationery shortage by making sure I supply facilities with reporting tools in time”*
			• *“[I am] responsible for all data for programs”*
Data quality	Responses related to data timelines, accuracy, as well as completeness, and general improvement in data quality	31	• *“All facilities are reporting and almost all are reporting on time”*
			• *“We used to have less than 50% facilities reporting and currently more than 90% of facilities are reporting on weekly notifiable diseases”*
			• *“There had been double reporting for [District X] and [District Y] but together with M*&*E Officers at [District Y] we managed to solve the problem”*
			• *“I managed to follow-up the facilities and explained the variables to the Officers; since then their reports have been correctly filled, almost every time”*
			• *“[We] conducted data audits…which helped the staff at facility level to better understand the variables in the forms and accuracy has improved”*
Data use	Conducting analysis, presenting data at meetings, and using data for evidence-based planning	29	• *“[I was] able to assist the Matron and PHS [Public Health Specialist] in analyzing some statistics and identify trends such as patient flow in the district, diarrhea cases, and malaria cases; and presented them to management in graphical form”*
			• *“[I ] came up with summaries of monthly reports for last year which would make it easy for comparison throughout quarters and even facility to facility”*
			• *“[I am] assessing program implementation to ensure whether the intended goals are achieved*”
			• *“Before my arrival, most of the activities were not evidence-based; they were not addressing any need… my involvement in EBP [evidence-based planning] made a significant change”*
Capacity building	Conducting workshops and sensitization session related to M&E, provision of one-on-one mentoring, feedback, and support visit for health workers	26	• *“Together with PMTCT coordinator, [I] conducted a workshop on reporting tools and variables”*
			• *“[I] managed to successfully conduct a workshop for District Health Officers at senior management to introduce the concept of M*&*E. It was highly appreciated by all”*
			• *“[I] managed to organize a workshop for CBOs [community based organizations] and taught them how they can monitor and evaluate their activities. This has made a great change”*
			• *“Reporting Officers have started to understand why reporting and have a sense of ownership on their reports”*
Other activities	Program implementation, newsletter development	21	• *“Participating in the development of the condom distribution strategy booklet for my district”*
			• *“Production of the first ever district newsletter”*
Reporting	Paper-based and electronic reporting to the national level	10	• *“Documenting the activities held per quarter in the district”*
			• *“Reporting on time to MLG {the Ministry of Local Government] every quarter”*
Operations research	Activities beyond routine monitoring and evaluation to inform program implementation	5	• *“Conducting a Pre–World AIDS day mini-survey on the community awareness of National Program and other interventions in regard to HIV AIDS in the district”*
			• *“[I was] part of the team that conducted the survey/study on the community awareness of the HIV/AIDS programs and interventions”*
			• *“I designed and administered a needs assessment survey in [District X] and surrounding areas which also helped in the EBP [evidence based planning] in terms of providing qualitative information/data”*

### Focus group discussions

Focus group discussions provided information on the district M&E Officers’ perspective of the training and mentoring activities. Participants reported that they preferred trainings with fewer participants, as these allowed for more one-on-one guidance. They also felt that trainings were most useful when they focused on practical information that directly related to their day-to-day responsibilities in the field. They suggested that the trainings needed more balance between basic didactic information provided to build a solid understanding of M&E and practical information the Officers needed to carry out their jobs. Weaknesses noted included insufficient training time for the amount of material and the need for more practical activities. When asked about additional training needs, participants from both focus groups requested training in advanced M&E topics to further build their expertise.

Officers reported that the mentoring provided an opportunity for individualized support in which they could raise important issues, solve problems, and improve their work. It also provided an opportunity to discuss their training needs and receive feedback. During each focus group, participants reported that the mentoring was beneficial, saying such things about their mentoring sessions as: “*they help us to stay focused*”, “*they aid in personal growth*”, “*they care, and [they] motivate us in our work*”, and “*they help in a lot of things related to data management*”. Suggestions for improving the mentoring component of the project included increasing the duration and frequency of the mentoring visits and ensuring that the mentoring schedule is communicated well in advance of the visit. Additionally, the Officers expressed interest in receiving progress reports based on the mentoring visits.

### Attrition assessment

Attrition was also a challenge for the new cadre. Six of the 51 district M&E Officer posts were vacated during the first 12 months (6/51; 12%). When asked about their reasons for leaving, the Officers expressed a variety of concerns, including inadequate support from supervisors, insufficient salary, high workload, and limited job security due to contract employment status. There were also complaints related to the limited professional progression opportunities.

## Discussion

These data indicate that the development of a cadre of district M&E Officers has contributed positively to the health information system in Botswana. In the absence of tertiary training in health informatics or M&E, on-the-job training and mentoring of university graduates can be an effective approach for developing a new professional cadre of M&E expertise and for strengthening capacity within a national health information system. The district M&E Officers initiated activities to strengthen data management, quality, reporting, and utilization for evidence-based planning. They also played an important role in raising awareness of health informatics and building M&E capacity among health workers and facility staff. While multiple studies have shown that task shifting can be an effective strategy for strengthening the provision of health services [1,2,22], there is less documentation in the literature related to the shift of health information responsibilities from clinicians to a non-clinical professional cadre as an approach to improving health information systems.

This initiative provides valuable information related to task shifting and the development of new professional cadres. One of the key lessons learned was the importance of ensuring job satisfaction. Twelve percent of the district M&E Officer posts were vacated during the first year, with low job satisfaction as the main factor. This is in-line with findings from a recent literature review of studies in Africa investigating the shifting of tasks from physicians to nurses that found that job satisfaction was closely related to staff retention and important for sustainability [23]. Given that the capacity development activities for this new cadre were based on the incremental building of skills, retaining staff was especially important. The attrition assessment highlighted the need to ensure clear roles and responsibilities, job security, and an appropriate career trajectory for this new cadre. These correspond to recommendations developed by WHO to guide task-shifting initiatives [1]. Another key lesson learned was that while it was important to help mitigate attrition, it was also important to plan for attrition by developing a strategy for efficient recruitment and development of training materials that could easily be used to train new staff. For example, the stakeholders guiding this project developed a set of self-guided workbooks that could be used as low-resource training materials to introduce new members of the cadre to health informatics and their day-to-day responsibilities [24].

There were multiple innovative aspects of this initiative. The approach addressed an urgent and immediate need for health informatics expertise through the provision of on-the-job training as opposed to long-term, off-site training. This on-going capacity building allowed for prompt strengthening of the health information workforce. Another unique aspect of this initiative is that while much of the initial funding was through PEPFAR, the initiative resulted in system-strengthening benefits that extend beyond the HIV/AIDS program as the district M&E Officers provided support to all district-based health programs. Training and mentoring, which are resource-intensive activities, were key components of this initiative. Therefore, there was a strong focus on country ownership and sustainability from the planning stages of the project that extended throughout the development of the cadre, with a focus on ensuring that the new cadre was a government-led initiative supported collaboratively by multiple stakeholders. This exemplifies the importance of country ownership. Subsequent to the development and implementation of this project, country ownership has been recommended internationally as an important approach to donor assistance [25]. While the posts for the M&E Officers were initially provided by donor funding through fixed-term contracts, steps have been initiated for absorption of the cadre into the Government of Botswana public health workforce.

A skilled health information workforce is a critical component of a well-functioning national health system [26]. This study emphasized the importance of capacity development as a means to improve data quality. While these data indicate that the M&E Officers gained knowledge and skill in certain areas, a significant change in these competencies resulting from further training and mentoring related to areas such as basic epidemiology, program evaluation, and self-assessment was not detected. The importance of capacity building to strengthen health systems is supported by data from a study in South Africa that found that training health information personnel and program managers in data collection, in conjunction with monthly data reviews and data audits, improved the completeness and accuracy of data for monitoring the PMTCT program [10]. It is, however, important to note that the challenges affecting health information systems are complex and multifactorial and that capacity development initiatives must be implemented alongside other system-strengthening initiatives to have the most substantive impact. This would include initiatives to implement electronic data management systems [9,17], instill a culture of data use [27], and harmonize data collection efforts [17,26,28,29].

A limitation of the data presented in this article is that they were collected for routine monitoring of the cadre as it was developed. The data related to M&E Officers’ skill level were based on self-reported measures and may not have reflected their actual skill level. There was a 12-month period of time between the initial skills assessment and the follow-up assessment. Factors other than the training and mentoring activities, such as self-efficacy or self-directed learning experiences, may have contributed to the improved skill levels reported, but these were not assessed or used to adjust the analyses. While these data provide important descriptive information regarding the system-strengthening activities initiated by the district M&E Officers, there is a need for further investigation of the impact of the cadre on health information systems after they have been in the field for a longer period of time to objectively examine data quality. Future assessment should include interviews or focus group discussions to provide more detailed information related to the achievements of the cadre. There was fluctuation within the population of district M&E Officers as some individuals did leave their posts over the 2 years in which the data were collected, while others were recruited into the cadre throughout the duration of the project.

The HIV/AIDS epidemic has placed a burden on health information systems as the amount of and demand for data have increased with the escalation in care, treatment, and prevention activities. Sound M&E systems are more important than ever because of resource constraints that necessitate evidence-based decision-making in order to target limited resources as efficiently as possible [17], performance-based funding mechanisms implemented by some major funders [30], and the emphasis on monitoring Millennium Development Goals [31]. While achieving data quality has been shown to be a challenge, even in non-resource limited settings [32,33], reports from countries such as Uruguay suggest that high data quality can be achieved in resource-constrained environments [34]. The timely availability of quality data is crucial for addressing health challenges and improving the quality of care throughout the health system. Initiatives such as the one presented in this article are needed to ensure adequate human resources to support national health information systems.

## Conclusion

The development of a cadre of district M&E Officers has contributed positively to the health information system in Botswana through the initiation of a variety of activities to strengthen data management, quality, reporting, and utilization for evidence-based planning. In a context where a pool of trained health informatics personnel is not available, university graduates can be recruited to participate in intensive on-the-job training and mentoring programs, a successful approach to strengthening health information systems.

## Competing interests

The authors declare that they have no competing interests.

## Authors’ contributions

JHL participated in the development and implementation of the district M&E Officer cadre, study design and data collection, data analysis, and manuscript writing. LR participated in data collection, data analysis, and manuscript writing. SMB, LB, SB, EM, SM, MM, and BWS participated in the implementation of the district M&E Officer cadre, data collection, data analysis, and revision of the manuscript. RL and SL participated in the development of the district M&E Officer cadre and the revision of the manuscript. BS participated in the development of the district M&E Officer cadre, data analysis, and manuscript writing. All authors read and approved the final manuscript.
